# Complications in image-guided musculoskeletal injections

**DOI:** 10.1007/s00256-022-04076-8

**Published:** 2022-05-27

**Authors:** John P. Hynes, Eoin C. Kavanagh

**Affiliations:** Department of Radiology, National Orthopaedic Hospital Cappagh, Finglas, Dublin 11, Republic of Ireland

**Keywords:** Musculoskeletal interventions, Complications, Sports medicine, Tendon rupture

## Abstract

Complications in musculoskeletal interventions are rare and where they do occur tend to be minor, and often short-lived or self-limiting. Nonetheless, the potential for significant complications exists, and a thorough understanding of both the mechanisms which contribute and the manner in which they may clinically present is of critical importance for all musculoskeletal radiologists involved in performing procedures, both to mitigate against the occurrence of complications and to aid rapid recognition. The purpose of this review is to analyse the relevant literature to establish the frequency with which complications occur following musculoskeletal intervention. Furthermore, we highlight some of the more commonly discussed and feared complications in musculoskeletal intervention, such as the risk of infection, potential deleterious articular consequences including accelerated joint destruction and the poorly understood and often underestimated systemic effects of locally injected corticosteroids. We also consider both extremely rare but emergent scenarios such as anaphylactic reactions to medications, and much more common but less significant complications such as post-procedural pain. We suggest that meticulous attention to detail including strict adherence to aseptic technique and precise needle placement may reduce the frequency with which complications occur.

## Introduction and background

The utilization of image guidance in musculoskeletal intervention is an important and now widespread skill which is fundamental to both diagnostic and therapeutic purposes across a broad range of musculoskeletal and rheumatological conditions. Accurate targeting of both joints and related extra-articular structures including bursae, tendons and sites of nerve entrapment is critical to symptom relief, allows fluid sampling and analysis for diagnostic purposes and facilitates enhanced cross-sectional imaging techniques in the form of computed tomography or magnetic resonance (MR) arthrography.

### Classification and incidence of complications

Complications—adverse events arising following a procedure—are generally thought of in terms of those either occurring locally in the region of the injection site, or systemic effects occurring remotely which are usually attributed to the effects of the injectate [1]. They are also categorised based upon their severity and the resulting additional care required, according to the classification of adverse events described by the Society of Interventional Radiology as follows [2]:

- Mild: no or nominal therapy required
- Moderate: moderate care escalation requiring significant additional treatment, e.g. unplanned overnight admission, blood product administration or prolonged outpatient follow-up
- Severe: marked escalation of care involving prolonged inpatient admission, complex intervention requiring general anaesthesia or intensive care admission
- Life-threatening or disabling events: cardiopulmonary arrest, shock, organ failure, dialysis, paralysis, loss of limb or organ
- Patient death or unexpected pregnancy abortion.

In general, complications—particularly those classified as moderate, severe, or resulting in disabling events or death—are uncommon following image-guided musculoskeletal injection. It is difficult to establish precisely the frequency with which complications occur due to heterogeneity in how these are recorded and reported in the literature. For example, a previous large study of over 8000 patients had only a solitary major complication which occurred following a spinal procedure, with no major complications recorded in the non-spinal cohort [1], and an overall incidence of adverse effects of 1%. The recording of complications however relied on the patient either contacting or attending the department at which they had undergone injection, with potential for underreporting. A systematic review of extra-articular corticosteroid injections reported minor adverse events in 0–81%, with major adverse events in 0.5–8% [3]. A Cochrane analysis of image-guided shoulder injections reported adverse events in 18.1%, none of which were serious [4]. While the exact risk is difficult to ascertain, the potential for untoward consequences is something that should be considered by all practitioners involved in the provision of such procedures, in order to both mitigate against their occurrence and aid swift recognition where they do materialize.

### Image guidance and targeted structures in musculoskeletal procedures

Ultrasound, fluoroscopy and CT are the most commonly utilized modalities in targeted injection. Image guidance provides superior, more accurate needle placement in comparison to traditional ‘blind’ or ‘landmark-guided’ techniques [5] and should also mitigate against the incidence of complications occurring locally at the injection site due to the ability to identify and avoid important regional neurovascular structures.

Intra-articular injections are one of the most commonly performed musculoskeletal interventions, with the shoulder, elbow, wrist, hip, knee and ankle all representing common targets. Injections are typically performed for a combination of diagnostic and therapeutic indications. The alleviation of pain may fulfil both criteria, by confirming that a particular joint is the source of a patient’s symptomatology and providing lasting relief. The aspiration of fluid from any joint allows analysis including for example microscopy and culture to confirm or exclude the presence of septic arthritis, or polarized light microscopy examination to identify crystals suggestive of gout or CPPD (calcium pyrophosphate dihydrate deposition). Intra-articular injection of iodinated contrast medium or dilute gadolinium solutions enables detailed evaluation of intrinsic structures at CT and MR arthrography, in particular the glenoid labrum of the shoulder and acetabular labrum of the hip. The specific approach to a particular joint and the choice of imaging guidance depends partly on operator preference and partly on the specific indication for the procedure. Extra-articular injections targeting important musculoskeletal structures, particularly peritendinous and intrabursal injections, are also frequently encountered. The sub-acromial bursa, medial and lateral epicondyles, carpal tunnel, trochanteric bursa, patellar tendon and plantar fascial origin are just some of the more routinely targeted sites. Again, the specific choice of imaging modality is operator and indication-dependent; however, in general, ultrasound is most commonly utilized due to the soft tissue resolution provided and the ease of real-time needle guidance.

### Injectates

The choice of injectate is important and may affect both the incidence and nature of complications. Synthetic corticosteroids (in combination with local anaesthetic) are the mainstay of treatment in image-guided musculoskeletal interventions, a practice established over 70 years ago [6]. They act primarily by affecting cytokine activity and are powerful anti-inflammatory agents. Corticosteroid preparations for injection may be soluble (such as dexamethasone) or insoluble, forming microcrystalline suspensions (such as methylprednisolone or triamcinolone) [7]. Local anaesthetics are administered both to the superficial tissues, to provide the patient comfort, and at the injection site itself—intra- or extra-articular—to allow post-procedural relief. They inhibit nerve transmission temporarily by binding sodium channels, producing local anaesthesia [8]. As with corticosteroid preparations, there is an array of local anaesthetic formulations in common clinical use, the specific properties of which are beyond the scope of this article. Clinically, the onset and duration of action are the principal factors in determining which is used in specific settings. The choice of local anaesthetic used in musculoskeletal intervention varies between providers; our practice typically uses bupivacaine, which is more potent and has a longer duration of action than the less potent lidocaine; however, lidocaine displays a more rapid onset of action [9]. In recent years, concerns have been raised regarding the potential chondrotoxicity of bupivacaine which may influence this decision [10].

Less commonly, but increasingly, hyaluronic acid may be used in intra-articular injections, specifically for the treatment of osteoarthritis. Hyaluronic acid is a viscoelastic entity which occurs naturally in connective tissues and synovial fluid. It is postulated to act as a shock absorber and lubricant when administered via intra-articular injection, most commonly to the knee or hip joints [11]. Another emerging treatment in musculoskeletal intervention is the use of platelet-rich plasma (PRP), which has been applied for a variety of conditions but most notably tendinopathy [12]. PRP injection involves autologous blood harvesting, centrifugation of the sample to produce a platelet-rich specimen and subsequently administering this via ultrasound-guided injection. The aim is ultimately to stimulate the healing process via the release of growth and differentiation factors. Needle fenestration of the tendon may be performed concurrently [13].

### Complications in image-guided musculoskeletal injection

#### Local complications following intra-articular injections

##### Infection and mimics

**Fig. 1:**
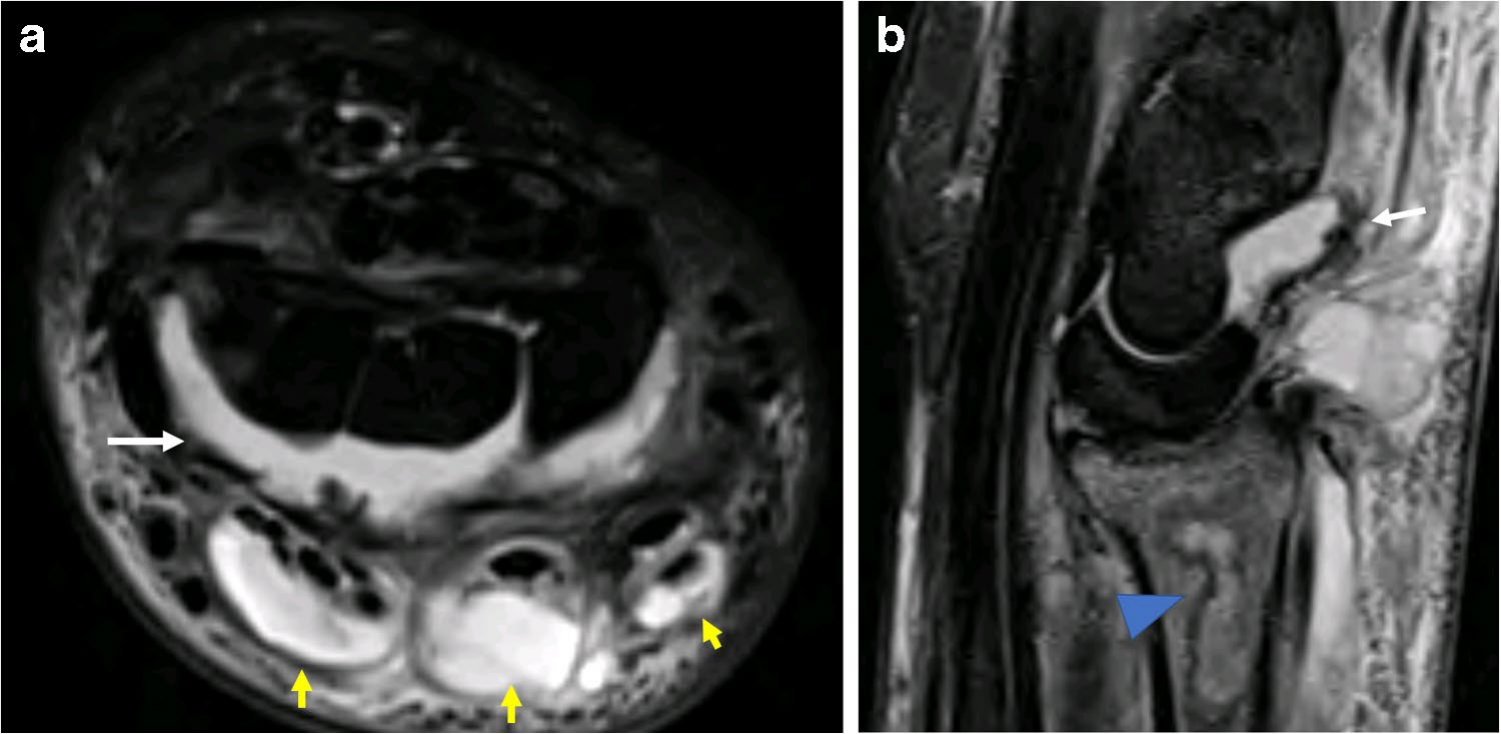
MRI of the right wrist in a 45-year-old man who presented with severe wrist pain, erythema and swelling 5 days after a fluoroscopic-guided wrist corticosteroid injection. Joint aspiration subsequently confirmed septic arthritis. Axial (**a**) and sagittal (**b**) STIR images of the right wrist demonstrate large radiocarpal and intercarpal effusions (white arrow), with marked changes of multifocal extensor tenosynovitis (yellow arrows). There is geographic signal abnormality in the distal radius in (**b**) consistent with osteomyelitis (arrowhead)

**Fig. 2:**
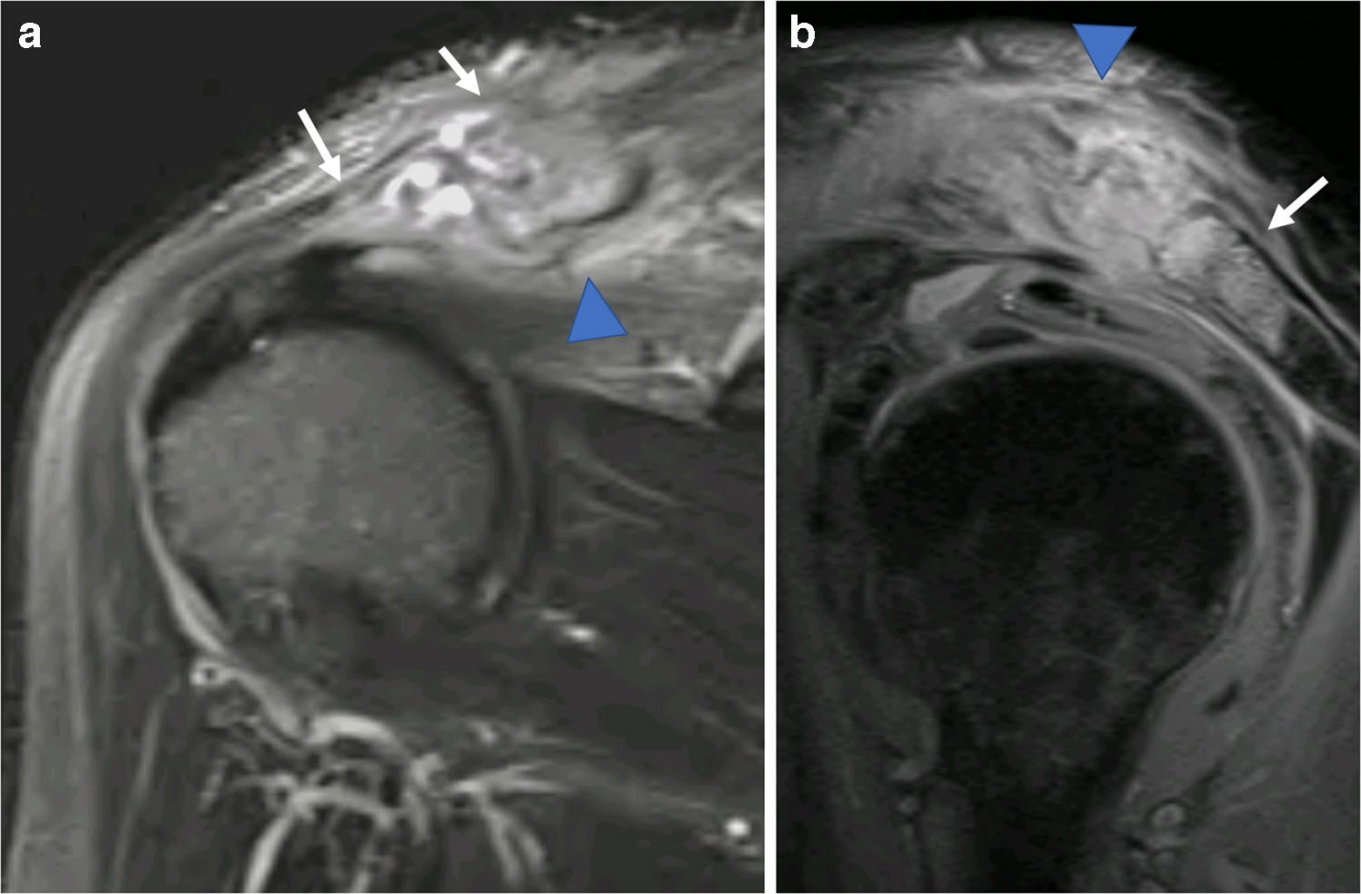
MRI of the left shoulder in a 65-year-old woman who presented with shoulder pain and fever 1 week after an ultrasound-guided acromioclavicular joint corticosteroid injection. Joint aspiration subsequently confirmed septic arthritis. Coronal (**a**) and sagittal (**b**) STIR images of the left shoulder demonstrate florid bone oedema spanning the acromioclavicular joint (white arrows), with marked periarticular soft tissue and subcutaneous oedema (arrowhead)

Other than immediately life-threatening events such as anaphylaxis, infection—in particular septic arthritis following an intra-articular injection—is potentially the most devastating and therefore most feared musculoskeletal complication (Figs. 1 and 2). It is relatively rare in the literature; however, multiple reports do exist. This reinforces the importance of meticulous attention to sterile technique, aseptic skin preparation and sterile draping as *Staphylococcus aureus* is the commonest causative organism, and contamination of the needle with the patient’s skin flora is the presumed mechanism by which the inadvertent seeding of the joint occurs. The Society of Interventional Radiology in its guidance on the requirement for antibiotic prophylaxis around interventional procedures regards musculoskeletal interventions as ‘clean’, and routine antimicrobial use is not recommended [14].

A less destructive condition which may mimic septic arthritis following an intra-articular injection is transient sterile chemical synovitis [15]. This condition is widely referred to as ‘the flare reaction’, although in truth this term is widely and interchangeably used to describe a multitude of symptoms and signs following an injection—ranging from a simple increase in perceived pain, to a more concerning syndrome of severe pain, swelling, erythema and joint effusion which can genuinely mimic an infected joint [16]. It may be extremely difficult to differentiate between significant transient synovitis and septic arthritis, as the clinical presentation may be so similar inflammatory markers may be elevated in both entities. When this is the case, joint aspiration is required to confirm the absence of organisms at culture with antibiotic cover in the interim. While this constellation of symptoms may be concerning to patient and clinician alike, flare reactions are for the most part self-limiting. Some evidence suggests that this type of reaction occurs more commonly following injection of viscoelastic supplements, possibly due to their higher molecular weight [17, 18]. A similar entity which may mimic steroid flare is the induction of acute calcium pyrophosphate dihydrate arthritis following intra-articular viscosupplementation which has been described, manifesting as severe pain, swelling and impaired function [19]. These cases typically resolve with relatively simple measures including oral non-steroidal anti-inflammatories (NSAIDs) [20].

A related consideration for the musculoskeletal radiologist is the relative responsibility of the radiologist versus the referring clinician in patient follow-up and management of complications. This balance is likely to vary substantially between different institutions depending on departmental practices and local arrangements. Our practice is to facilitate the initial assessment where a patient contacts the department describing a potential adverse event, but to defer to the referring clinician thereafter, or arrange further specialist referral as required. This achieves the balance of prompt assessment and recognition of possible complications while ensuring the patient is cared for by the most appropriate clinician going forward. We do not arrange routine follow-up post injection; patients typically have an appointment for follow-up with their referring clinician.

##### Adverse joint events

In recent years, increased attention has been paid to the potential for adverse joint events following intra-articular injection, including osteonecrosis, subchondral insufficiency fracture and rapidly progressive osteoarthritis [21]. Patients undergoing corticosteroid injection for the management of hip osteoarthritis appear to be at increased risk; however, the picture and our understanding of the patient characteristics involved remain incomplete. Both corticosteroids and the local anaesthetic formulations in combination with which they are typically administered may have deleterious effects upon human chondrocytes. The precise mechanisms by which this occurs are complex; however, corticosteroids appear to adversely affect cartilage production and maintenance by their impact upon important proteins including proteoglycan and collagen [22]. The chondrotoxic effects of local anaesthetics appear to act in dose and time-dependent ways [23]. The fear is that this leads to an acceleration of the osteoarthritis for which the patient was referred, with rapidly progressive osteoarthritis (RPOA) having been described by several authors [24]. This entity lacks a clear, widely accepted definition but constitutes an accelerated, destructive arthropathy which is typically painful and may be confused with other processes due to its often striking appearances at imaging. Two types have been described, RPOA type 1 which is associated with progressive and severe joint space narrowing in the absence of significant bone loss and RPOA type 2 which constitutes rapid destruction of the joint with accelerated bone loss [25]. Two additional interrelated conditions are subchondral insufficiency fracture (SIF) and osteonecrosis. Subchondral insufficiency fractures are typically seen in weight-bearing areas, presenting as acute pain in the absence of a history of trauma. Subchondral insufficiency fracture may be radiographically occult; however, it is important to consider as a potential cause of pain prior to intra-articular corticosteroid injection as steroids may adversely affect the healing process, and this may potentially contribute to progression to fragmentation and ultimately collapse of the articular surface [26]. Osteonecrosis refers to the ischaemic death of osseous constituents, and intra-articular corticosteroid injection has been implicated also in this process, both due to potential negative effects of steroids on osteoblastic function and also the propensity for increasing marrow fat which may in turn adversely affect bone perfusion [21, 27]. The precise relationship between intra-articular corticosteroid injections and the progression of arthritis/subchondral insufficiency fracture/osteonecrosis is complex and difficult to fully elucidate with multiple potential confounding factors; however, the potential implications warrant consideration and caution when contemplating injection. This is an area that undoubtedly deserves further attention, and greater insight would be warmly welcomed.

#### Local complications following extra-articular injections

Image-guided corticosteroid injection is also utilized in the management of a variety of extra-articular conditions, including pathologies affecting tendons, ligaments, entheses, bursae and certain entrapment neuropathies. This creates the potential for additional locally mediated adverse effects, including altered skin pigmentation, atrophy of the subcutaneous tissues, infection and tendon or fascial rupture.

##### Tendon rupture and local tissue effects

**Fig. 3:**
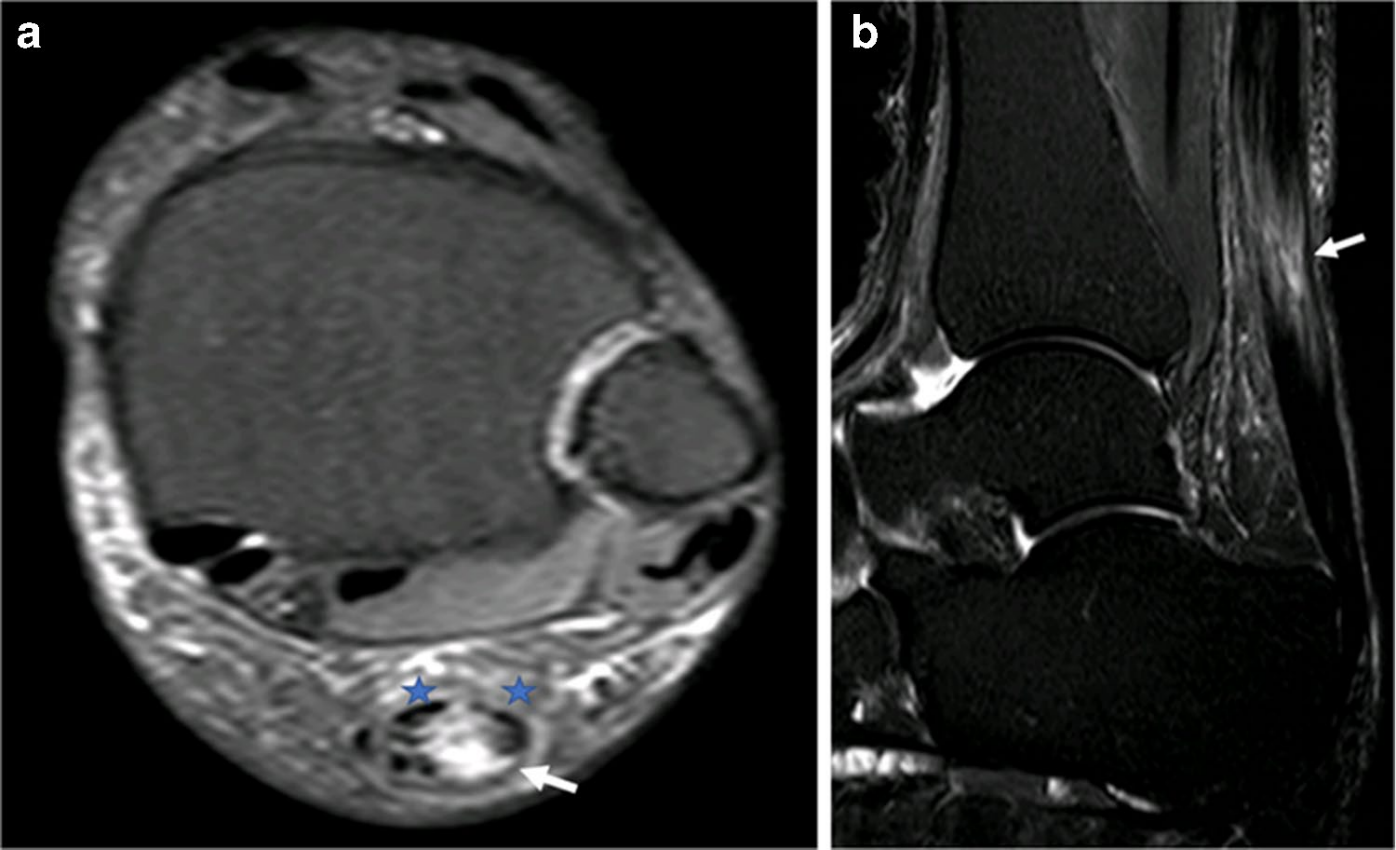
MRI of the left ankle in a 51-year-old woman who experienced acute onset severe pain in the distal calf 4 days after an ultrasound-guided peritendinous Achilles corticosteroid injection. Axial (**a**) and sagittal (**b**) STIR images of the left ankle demonstrate near complete rupture of the Achilles tendon (arrows) at the musculotendinous junction. Some peripheral fibres remain intact preventing total discontinuity (stars in (**a**))

Tendon rupture has been described following steroid injection [3, 28] and can have significant consequences, particularly in an active or athletic cohort. In high-level elite and professional athletes, this may result in adverse career prospects or loss of income. It is difficult if not impossible to imply causality despite the number and variety of reported cases, and there is some evidence that the true frequency of tendon rupture following a corticosteroid injection is lower than sometimes is anecdotally reported [29]. The abnormality of the tendon that prompted the injection for example can be implicated, and there is a suggestion that the incidence of tendon rupture increases with the number of injections undergone by the patient which may equally reflect worsening native tendon abnormality, the accumulative effect of multiple injections or a combination of these factors [30]. Injection technique must be considered given the potentially deleterious mechanical effects of intratendinous needle placement. There is some evidence that the choice of corticosteroid may affect the incidence of tendon rupture following injection, with triamcinolone acetonide being associated with a higher incidence of rupture than betamethasone, methylprednisolone acetate or hydrocortisone [30]. Post-injection tears of the patellar, quadriceps, Achilles and biceps tendons are those most frequently encountered in the literature (Fig. 3). Less commonly described are tears of the common extensor origin at the lateral epicondyle, the supraspinatus, tibialis anterior, biceps femoris, triceps and the small flexor tendons of the hand. Plantar fascia rupture may also be encountered and was commoner than any individual tendon rupture in one large review of complications following injection for athletic injury [30].

Both hypo- and hyperpigmentation of the skin have been described following steroid injection, as has atrophy of the fat and subcutaneous tissues.

##### Infection 

Infection of the skin, soft tissues and even underlying osseous structures has been encountered following extra-articular injections [3], again highlighting the critical importance of meticulous adherence to sterile technique and skin preparation. Bursal injections appear to be associated with an extremely low incidence of complications with very few complications encountered in the literature; however, one of those was an ultimately fatal incidence of necrotising fasciitis [31]. Necrotizing fasciitis has also been described following steroid injection for trigger finger [32]. Osteomyelitis of the calcaneus and humerus have been described following injections for plantar fasciitis and lateral epicondylitis respectively [3]. Case reports also exist describing staphylococcal aureus abscess after an injection for Achilles tendinitis [33] and atypical mycobacterium infection following injection for De Quervain’s tenosynovitis [34].

##### Pain

To a degree, a certain amount of pain must be expected following an injection—as with any invasive procedure, despite attempts to mitigate this by various methods including the administration of local anaesthetic. Nonetheless post-procedural pain must be considered a complication as it meets the definition according to Edwards and Aronson—‘an appreciably harmful or unpleasant reaction, resulting from an intervention related to the use of a medicinal product, which predicts hazard from future administration and warrants prevention or specific treatment, or alteration of the dosage regimen, or withdrawal of the product’ [35]*.* With this in mind, post-procedural pain constitutes by far the commonest complication following corticosteroid injection, accounting for up to 58% of recorded complications [1]. While more serious entities including flare but particularly infection must be excluded, in the majority of instances, this is attributable to the local mechanical effects of both needle and injectate, exacerbating symptoms in a patient population predominantly already experiencing significant levels of pain. Post-injection pain in the absence of more sinister underlying causes is generally benign and self-limiting, resolving with simple analgesia. The fact that post-injection pain appears to account for at least half, if not more, of complications would seem to suggest a possible role for the utilization of prophylactic analgesia in an attempt to mitigate against this.

#### Systemic complications

##### Steroid-mediated systemic sequelae

The systemic side effects associated with both intra- and extra-articular corticosteroid injections remain incompletely understood. The systemic absorption of corticosteroid occurring following a local injection is variable and may be underestimated [36]. The adverse effects of systemically administered glucocorticoids are manifold and beyond the scope of this article, including but not limited to endocrinological, immunological, cardiovascular, osseous and neuropsychiatric sequelae. Any of these effects may be seen in association with locally injected corticosteroids; however, they are of an order of magnitude less than in the context of systemic administration. This is because in the vast majority of instances—particularly where there is accurate intra-articular administration—systemic absorption of locally injected musculoskeletal corticosteroid is miniscule [37].

Several factors are thought to impact the degree to which locally injected corticosteroids are absorbed. Less soluble corticosteroid formulations, such as triamcinolone and methylprednisolone acetate, appear to deliver a greater and more prolonged suppression of endogenous serum cortisol than more soluble corticosteroid preparations such as betamethasone [38]. Patients who are concomitantly receiving certain medications, in particular cytochrome P450 3A4 inhibitors such as ritonavir, appear to be at risk of suffering very severe and prolonged systemic side effects attributable to corticosteroids—including Cushing’s syndrome and adrenal suppression—following even a single intra-articular injection [39, 40]. While not specifically affecting absorption, cytochrome P450 3A4 inhibitors appear to significantly reduce the clearance of administered corticosteroids from the patient’s system, and extreme caution is recommended in these patients.

The systemic side effects outlined above are very much towards the more extreme end of the spectrum. However, even very tiny amounts of systemic absorption following local corticosteroid injection may account for the more commonly observed and minor effects attributed to corticosteroids following injection. Facial flushing is commonly experienced following corticosteroid injection, postulated to occur secondary to a histamine-mediated response, and most patients are counselled to expect this post-procedure. A perhaps underappreciated systemic effect is an increase in serum glucose levels in diabetic patients who have undergone corticosteroid injection, which has been demonstrated in several studies [41, 42]. Additional ‘constitutional’ side effects which are often attributed to systemic absorption of injected corticosteroid include headache and gastrointestinal disturbance. In general, these are mild and self-limiting or resolve with simple measures [7].

##### Anaphylaxis

As with any situation in which a medication is administered, the potential for anaphylaxis must be considered; however, this is extraordinarily rare in the setting of corticosteroid injection with only a handful of case reports describing it [43]. This may occur secondary to the steroid itself or, more commonly, to polyethylene glycol (macrogol) which acts as a solvent for particulate corticosteroids [44]. Access to emergency medications including adrenaline is therefore an important consideration for practitioners providing corticosteroid injections, despite the relative rarity with which this occurs. Knowledge of the emergent management of this uncommon entity is a prerequisite.

## Conclusion

In conclusion, image-guided musculoskeletal injections are safe procedures in general. Complications are uncommon and tend to be minor and short lived in those instances where they do occur. Major complications are extremely rare but, where occurring, may have significant consequences for the patient. A thorough understanding of the mechanisms by which complications may arise is essential for musculoskeletal radiologists involved in performing injections. This allows mitigation against the occurrence of complications and enables swift recognition and prompt action where they do occur.
